# Real-world experience of adverse reactions-necessitated rifampicin-sparing treatment for drug-susceptible pulmonary tuberculosis

**DOI:** 10.1038/s41598-023-38394-1

**Published:** 2023-07-12

**Authors:** Hyung-Jun Kim, Ye Jin Lee, Myung Jin Song, Byoung Soo Kwon, Yeon Wook Kim, Sung Yoon Lim, Yeon-Joo Lee, Jong Sun Park, Young-Jae Cho, Choon-Taek Lee, Jae Ho Lee

**Affiliations:** 1 Division of Pulmonary and Critical Care Medicine, Department of Internal Medicine, Seoul National University Bundang Hospital, Seongnam, Republic of Korea; 2 Department of Internal Medicine, Seoul National University College of Medicine, Seoul, Republic of Korea

**Keywords:** Tuberculosis, Respiratory tract diseases

## Abstract

Rifampicin is an important agent for tuberculosis treatment; however, it is often discontinued because of adverse reactions. The treatment regimen then can be administered as that for rifampicin-resistant tuberculosis, which can be toxic. We retrospectively reviewed 114 patients with drug-susceptible pulmonary tuberculosis who discontinued rifampicin due to adverse reactions during an 18 year period at a tertiary referral center, of which 92 (80.7%) exhibited favorable response. Hepatotoxicity was the leading cause of intolerance. Patients with a favorable response were younger and less likely to have comorbidities. The majority of patients were administered four medications during the intensive phase and three to four during the consolidative phase. For those with a favorable response, the median duration of treatment was 10.2 months and the most common intensive regimen was a combination of isoniazid, ethambutol, pyrazinamide, and fluoroquinolone (25%). The most common consolidation regimen was a combination of isoniazid, ethambutol, and fluoroquinolone (22.8%). Among the patients with a favorable response, two (2.2%) experienced recurrence after a follow-up of 3.4 (interquartile range 1.8–6.8) years. For patients with drug-susceptible pulmonary tuberculosis who do not tolerate rifampicin owing to its toxicity, a shorter regimen may be a useful alternative.

## Introduction

Tuberculosis is an infectious disease caused by *Mycobacterium tuberculosis* and it has been the major cause of death caused by a single pathogen before the coronavirus disease 2019 pandemic^1^. Without treatment, the mortality rate of tuberculosis can range from 20 to 70%^2^. Fortunately, around 85% of patients can be cured using a standard regimen of isoniazid, rifampicin, ethambutol, and pyrazinamide^3^.

Among the first-line regimens against tuberculosis, rifampicin is one of the key players. Rifampicin is a bactericidal agent that interferes with the ꞵ-subunit of the RNA polymerase of *M. tuberculosis,* which shortens the duration of treatment and reduces the subsequent relapse rate^4^. However, a previous report showed that the discontinuation rate of rifampicin was approximately 1.9% due to its adverse reactions such as hepatotoxicity, skin reactions, and abnormalities in blood cell counts^5,6^. Such adverse reactions may limit further use of rifampicin during the treatment course.

However, the optimal treatment strategy after omission of rifampicin from the treatment regimen is not well established. When rifampicin cannot be used, a regimen used for patients with rifampicin-resistant tuberculosis can be used. World Health Organization suggests that these patients be treated similar to cases of multidrug-resistant (MDR) tuberculosis^7^. Despite recent reports of shorter regimens for MDR tuberculosis^8–11^, 18–20 months of treatment, including toxic drugs such as bedaquiline, linezolid, and cycloserine, is currently recommended as the standard practice in South Korea^12^. But these drugs are also associated with adverse events, such as peripheral neuropathy, skin reactions, and hepatotoxicity with an occurrence rate of 1.3–14.1%^13^. Alternatively, a tailored regimen of isoniazid, ethambutol, pyrazinamide, and fluoroquinolone for 12–18 months can be considered based on clinical experience^14^. However, such a regimen lacks solid scientific background; whereas recent studies suggest that a shorter regimen may be sufficient for drug-resistant tuberculosis^15,16^, and it may be advantageous over a longer regimen in terms of patient compliance.

In the real-world practice, rifampicin is usually included in the initial regimen unless otherwise contraindicated. Even if rifampicin is omitted during the treatment course owing to its adverse effects, short administration of this drug may be effective for patients with drug-susceptible tuberculosis. This differs from patients with rifampicin-resistant tuberculosis, in whom rifampicin is ineffective during the entire treatment course. In addition, while patients with rifampicin-resistant tuberculosis often have resistance to other first-line drugs, this is not necessarily the case for patients who cannot tolerate rifampicin. In this regard, we aimed to share the real-world experience of adverse reactions-necessitated rifampicin-sparing treatment among patients with drug-susceptible pulmonary tuberculosis.

## Methods

### Patient selection and baseline information

From October 1, 2003, to August 31, 2020, patients diagnosed with pulmonary tuberculosis were screened at a tertiary referral center in South Korea based on reported cases to the Korea Disease Control and Prevention Agency. Following the exclusion of MDR tuberculosis, we identified patients with pulmonary tuberculosis who exhibited intolerance to rifampicin due to adverse reactions through a review of electronic medical records. As our study was retrospective in nature, adverse reactions were not pre-defined. However, they were determined by the attending physician and the information was collected via medical records review. Adverse reactions included, but were not limited to, hepatotoxicity, skin reactions, and flu-like fever^17,18^. Patients with tuberculosis resistant to any anti-tuberculosis medications, those treated with rifampicin for > 6 months, those who were transferred out, those who were still under ongoing treatment, and those who had an unclear treatment history were excluded. Drug susceptibility was tested conventionally on Lowenstein–Jensen media using the absolute concentration method (Supplementary Appendix 1), except for pyrazinamide, for which the pyrazinamidase test was performed. We selected patients confirmed to be susceptible for all anti-tuberculosis medications.

Baseline patient data, including baseline demographics, underlying diseases, treatment regimens, and treatment outcomes, were extracted retrospectively from the electronic medical records system. The Institutional Review Board of Seoul National University Bundang Hospital (B-2204–750-106) approved this study and waived the requirement for informed consent due to the use of anonymized data and the study's retrospective nature. This study was conducted in accordance with the principles of the Declaration of Helsinki.

### Interpretation of the treatment regimen

Patients were treated in accordance with the local treatment recommendations,^12,19–21^ which remained mostly consistent throughout the study period. The treatment regimen could be modified with the liberty of the attending physician depending on the patient’s response to treatment. Anti-tuberculosis medications used during the treatment period were screened. The types of medications and their duration of use were inspected, and the duration of each drug was described in detail. Duration of each drug was calculated as actual dosage days prescribed, while the overall treatment duration was calculated by counting the calendar days from the first day of treatment to the last. Furthermore, a representative regimen was chosen, which was characterized as regimens that last for a minimum of 2 months^22^. The patients were predominantly subjected to two distinct treatment regimens. The initial regimen was deemed as the intensive regimen, while the subsequent regimen was considered as the consolidative regimen.

### Definition of initial treatment response

As the study was retrospective in nature, there were no pre-established protocols for conducting microbiological follow-ups subsequent to treatment. Therefore, patients were divided into two groups according to their treatment response: favorable and unfavorable. A favorable response was defined as being cured or completing the treatment according to the recent 2021 World Health Organization definition^23^, apart from the possibility of changing treatment regimen due to adverse reactions. An unfavorable response was defined as death during treatment, treatment failure regarding clinical and/or bacteriological response, or treatment abandonment including a loss to follow-up^23,24^.

### Follow-up after treatment completion

For patients who initially achieved a favorable response, we reviewed the electronic medical records using their unique hospital ID to determine if any recurrence of tuberculosis or death occurred after treatment. within addition to the medical records, sputum culture studies and chest X-ray results were also inspected. The date and primary cause of death were acquired from the death certificates prepared by the attending physicians and were confirmed using the Statistics Korea database with the Korean Standard Classification of Diseases, 7th edition, as of 31 December 2020. For those whose status could not be assessed using the above methods, we contacted them by telephone for their status as of May 10, 2022.

### Statistical analyses

Categorical variables are expressed as numbers (percentiles) using the chi-square test or Fisher’s exact test, whereas continuous variables are expressed as median (interquartile range [IQR]) using the Mann–Whitney U test. Odds ratios with 95% confidence intervals (CIs) were calculated using the logistic regression analyses. Statistical significance was set at *p*-value < 0.05. All statistical analyses were performed with Stata version 17.0 (StataCorp, College Station, TX, USA). The manuscript conforms to The Strengthening the Reporting of Observational Studies in Epidemiology Statement (Supplementary Appendix 2).

## Results

### Patient characteristics

**Figure 1:**
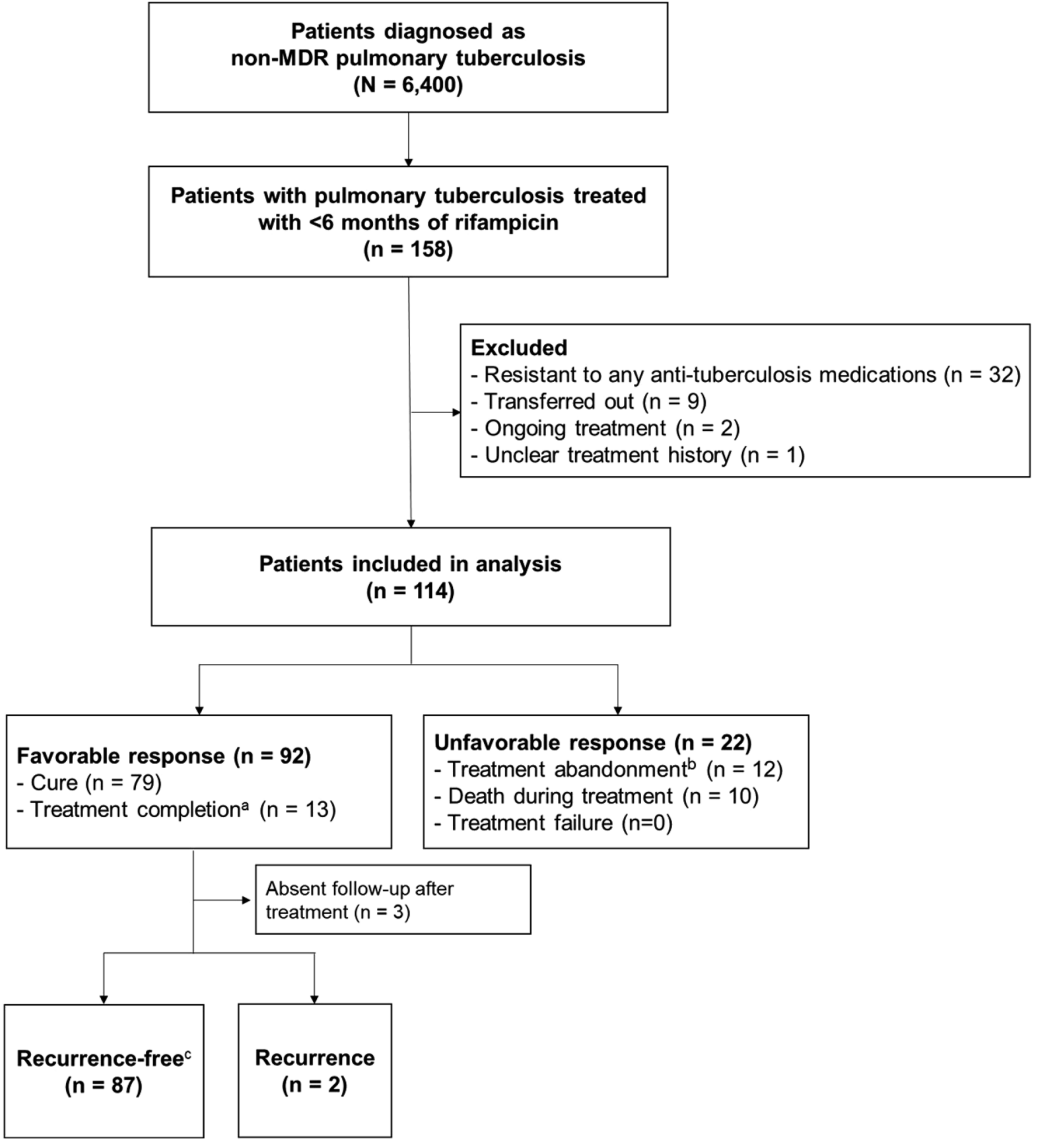
Flowchart of the patient selection process. ^a^Because some patients cannot expectorate further sputum after treatment, those who achieved clinical improvement and completed treatment according to the attending physician were included in the favorable response group. ^b^Includes patients who cannot continue treatment owing to underlying conditions (n = 9) and those who are lost to follow-up (n = 3). ^c^The duration of follow-up was a median of 3.4 (interquartile range 1.8–6.8) years. Abbreviation: MDR, multidrug-resistant.

During the study period, 6,667 patients were diagnosed with pulmonary tuberculosis and 267 patients were diagnosed with MDR tuberculosis. A total of 158 patients were unable to tolerate rifampicin due to adverse effects, of which 114 were included in the final analysis based on the selection criteria. Among 114 patients, 92 (80.7%) showed a favorable response to treatment. Among the patients in the favorable response group, 79 and 13 achieved cure and treatment completion, respectively. Among those in the unfavorable response group, treatment was abandoned in 12 patients either by the attending physician (n = 9) or the patient (n = 3), and the other 10 died during the treatment. Seven patients died due to infectious diseases possibly related to tuberculosis, while the other three of extrapulmonary malignancy (Fig. 1).

Patients with a favorable response were younger (median 61.0 [IQR 48.6–74.0] vs. 78.0 [IQR 70.0–80.0] years, *P* = 0.001), and were less likely to have comorbidities, such as diabetes mellitus (16.3% vs. 40.9%, *P* = 0.024) and chronic kidney disease (1.1% vs. 13.6%, *P* = 0.026). Cavities (25.0% vs. 59.1%, *P* = 0.002) and bilateral involvement (44.6% vs. 77.3%, *P* = 0.006) on chest radiographs were less common in patients with favorable responses. There were no significant differences with respect to sex (proportion of females: 40.2% vs. 27.3%, *P* = 0.379). Based on a multivariable analysis, it was determined that a younger age (adjusted odds ratio 0.94, 95% CI 0.90–0.99), the absence of chronic liver disease (adjusted odds ratio 0.05, 95% CI 0.01–0.60), and the absence of cavities (adjusted odds ratio 0.23, 95% CI 0.07–0.75) were significantly associated with a favorable response. Please refer to Supplementary Table 1 for further details.

Of the 114 patients, rifampicin was reintroduced once in 56 patients and twice in three patients. However, it was eventually excluded from the treatment regimen due to adverse reactions. Hepatotoxicity (59.6%) was the most common reason for discontinuation of rifampicin, followed by skin rash and/or pruritus (54.4%), gastrointestinal discomfort (50.9%), fever (28.9%), abnormal blood cell count (14.9%), headache and/or dizziness (13.2%), general weakness (9.6%), and renal injury (3.5%). Three patients did not use rifampicin in the first place due to expected hepatotoxicity regarding the underlying liver condition. The types of adverse reactions did not differ according to the initial treatment response (*P* > 0.050) (Table 1).

**Table 1:** Baseline characteristics of patients with tuberculosis who stopped rifampicin owing to adverse reactions.

Variables	Overall	Favorable response	Unfavorable response	*P*
	N = 114	n = 92	n = 22	
Age	66.5 [49.0–77.8]	61.0 [46.8–74.0]	78.0 [70.0–80.0]	0.001
Sex, female	43 (37.7)	37 (40.2)	6 (27.3)	0.379
BMI	21.1 [19.3–22.9]	21.2 [19.3–22.9]	20.6 [19.1–23.1]	0.604
Comorbidities				
Hypertension	27 (23.7)	19 (20.7)	8 (36.4)	0.201
Diabetes	24 (21.1)	15 (16.3)	9 (40.9)	0.024
History of tuberculosis	19 (16.7)	15 (16.3)	4 (18.2)	> 0.999
Malignancy	14 (12.3)	10 (10.9)	4 (18.2)	0.564
Chronic lung disease*	8 (7.0)	7 (7.6)	1 (4.5)	0.967
Chronic liver disease	5 (4.4)	2 (2.2)	3 (13.6)	0.075
Chronic kidney disease	4 (3.5)	1 (1.1)	3 (13.6)	0.026
Radiographic features				
Bilateral involvement	58 (50.9)	41 (44.6)	17 (77.3)	0.006
Presence of cavities	36 (31.6)	23 (25.0)	13 (59.1)	0.002
Adverse reactions to rifampicin				
Hepatotoxicity	68 (59.6)	55 (59.8)	13 (59.1)	> 0.999
Skin rash/Pruritus	62 (54.4)	51 (55.4)	11 (50.0)	0.825
Gastrointestinal discomfort	58 (50.9)	45 (48.9)	13 (59.1)	0.535
Fever	33 (28.9)	29 (31.5)	4 (18.2)	0.328
Blood cell count abnormality	17 (14.9)	14 (15.2)	3 (13.6)	> 0.999
Headache/Dizziness	15 (13.2)	13 (14.1)	2 (9.1)	0.782
General weakness	11 (9.6)	8 (8.7)	3 (13.6)	0.762
Renal injury	4 (3.5)	4 (4.3)	0 (0.0)	0.726

Numbers are presented as count (percentile) or median [interquartile range].

*Chronic lung disease refers to asthma, chronic obstructive pulmonary disease, and idiopathic pulmonary fibrosis. BMI, body mass index.

### Treatment regimen

Most patients started their treatment with the standard four-drug regimen (93.0%, isoniazid, rifampicin, ethambutol, and pyrazinamide) or three-drug regimen (2.6%, isoniazid, rifampicin, and ethambutol) (Supplementary Table 2). The attending physician subsequently prescribed a suitable intensive phase regimen for the patients, consisting of two to six drugs as outlined in Table 2. Out of the total of 114 patients, 66 were administered four or more medications, whereas 48 were administered three or fewer medications. In terms of achieving an initial favorable response to treatment, no statistically significant difference was observed between patients receiving four or more drugs (80.3%) and those receiving three or less drugs (81.3%) (*P* = 0.899).

**Table 2:** General information on the treatment regimen used for patients with tuberculosis.

Variables	Overall	Favorable response	Unfavorable response	*P*
	N = 114	n = 92	n = 22	
Treatment duration, months	9.3 [7.0–12.3]	10.2 [8.8–12.6]	3.8 [2.4–5.6]	< 0.001
Number of drugs for the intensive phase				0.797
4	60 (52.6)	49 (53.3)	11 (50.0)	
3	43 (37.7)	35 (38.0)	8 (36.4)	
2	5 (4.4)	4 (4.3)	1 (4.5)	
5	5 (4.4)	3 (3.3)	2 (9.1)	
6	1 (0.9)	1 (1.1)	0 (0.0)	
Number of drugs for consolidative phase				0.324
3	50 (43.9)	41 (44.6)	9 (40.9)	
4	48 (42.1)	38 (41.3)	10 (45.5)	
2	12 (10.5)	11 (12.0)	1 (4.5)	
5	4 (3.5)	2 (2.2)	2 (9.1)	
Drug combination for the intensive phase				0.040
1st line drugs* + Q	53 (46.5)	44 (47.8)	9 (40.9)	
1st line drugs* only	25 (21.9)	23 (25.0)	2 (9.1)	
1st line drugs* + Q + Other^‡^	24 (21.1)	14 (15.2)	10 (45.5)	
1st line drugs* + Other^‡^	8 (7.0)	7 (7.6)	1 (4.6)	
Q + Other^‡^	4 (3.5)	4 (4.4)	0 (0.0)	
Drug combination for consolidative phase				0.360
1st line drugs* + Q	60 (52.6)	50 (54.4)	10 (45.5)	
1st line drugs* + Q + Other^‡^	33 (29.0)	23 (25.0)	10 (45.5)	
1st line drugs* only	10 (8.8)	9 (9.8)	1 (4.6)	
Q + Other^‡^	6 (5.3)	6 (6.5)	0 (0.0)	
1st line drugs* + Other^‡^	5 (4.4)	4 (4.4)	1 (4.6)	

Representative regimens indicate regimens persisting for ≥ 2 months. Numbers indicate count (percentage) or median [interquartile range]. *First-line drugs indicate isoniazid, rifampicin, ethambutol, and pyrazinamide. ^‡^Drugs other than the first-line drugs or fluoroquinolones. Q, fluoroquinolone.

**Figure 2:**
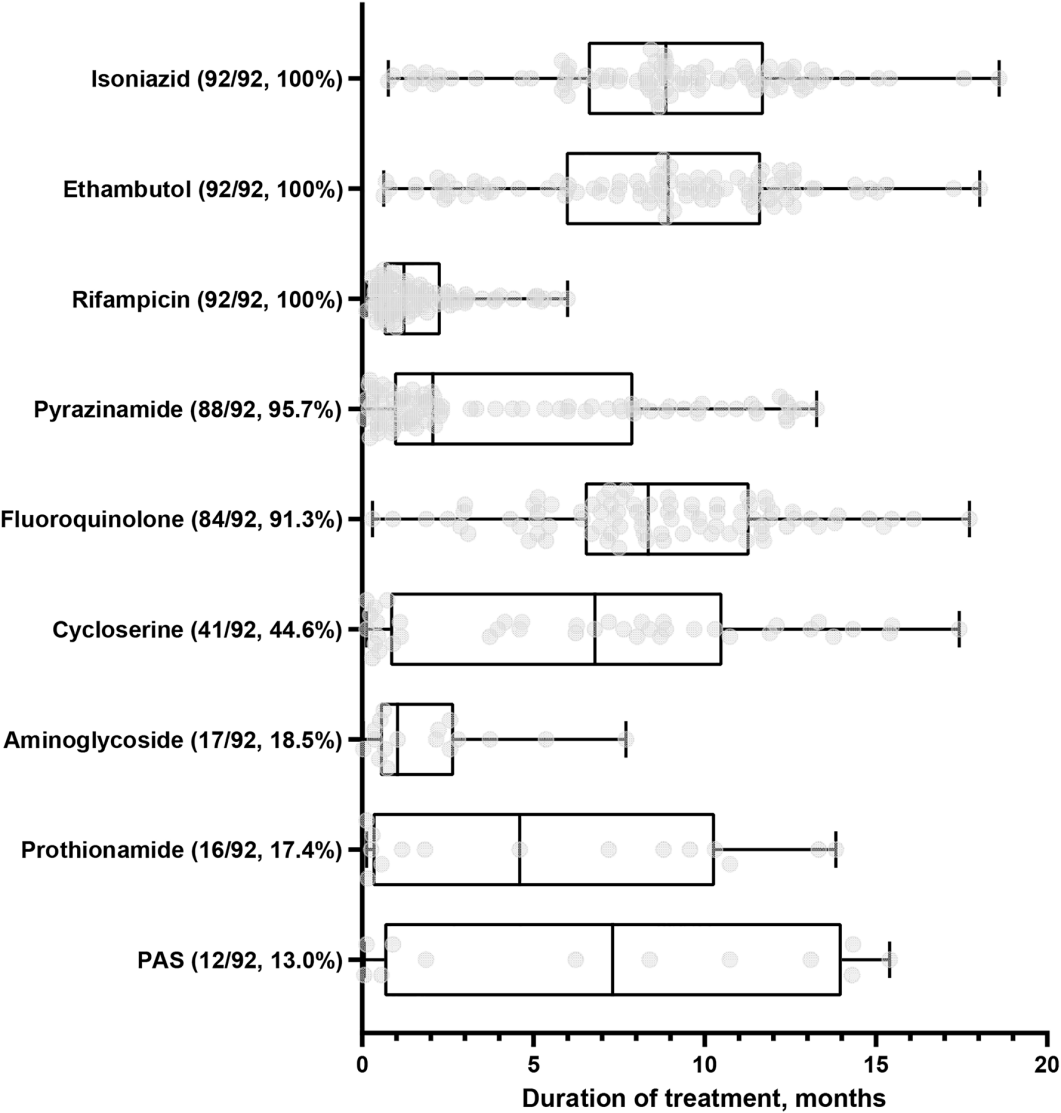
Duration of use of each anti-tuberculosis medication for the 92 patients with favorable outcomes. Box and whisker plots of 92 patients who show a favorable response upon initial treatment. The central line in each box represents the median. The box indicates the 25th and 7th percentiles, and whiskers indicate the range of the data. Gray dots indicate data for each patient. Abbreviations: PAS, p-aminosalicylic acid.

For the 92 patients with a favorable outcome, the median duration of treatment was 10.2 (IQR 8.8–12.6) months. During the intensive treatment phase, a four-drug (53.3%) or three-drug (38.0%) regimen consisting of first-line drugs with or without fluoroquinolone, was the most common. For the consolidation phase, three-drug (44.6%) or four-drug (41.3%) regimens consisting of first-line drugs with fluoroquinolone were the most frequently used (Table 2). The most common intensive regimen was a combination of isoniazid, ethambutol, pyrazinamide, and fluoroquinolone (25.0%), whereas the most common consolidation regimen was a combination of isoniazid, ethambutol, and fluoroquinolone (22.8%) (Supplementary Tables 3–4). Considering each drug, isoniazid (median 8.87 [IQR 6.65–11.73] months), ethambutol (median 8.93 [IQR 5.98–11.59] months), and rifampicin (median 1.22 [IQR 0.63–2.23] months) were used at least once in all patients. Pyrazinamide was used in 95.7% (median 2.07 [IQR 0.93–7.88] months) of patients. Fluoroquinolone was used in 91.3% (median 8.35 [IQR 6.63–11.28] months) of patients (levofloxacin in 45.7%, moxifloxacin in 35.9% and both in 9.8%). Other second-line medications included cycloserine (44.6%), aminoglycoside (18.5%), prothionamide (17.4%), and para-aminosalicylic acid (13.0%) (Fig. 2). Details are presented in Table 3.

**Table 3:** Use of each anti-tuberculosis drug and its duration according to the initial response to treatment.

Variables	Overall	Favorable response	Unfavorable response
	N = 114	n = 92	n = 22
Isoniazid	114 (100.0)	92 (100.0)	22 (100.0)
Duration, months	8.58 [4.67–11.27]	8.87 [6.65–11.73]	3.08 [1.30–4.73]
Fluoroquinolone	103 (90.4)	84 (91.3)	19 (86.4)
Duration, months	7.57 [4.63–10.55]	8.35 [6.63–11.28]	2.80 [1.00–3.38]
Ethambutol	113 (99.1)	92 (100.0)	21 (95.5)
Duration, months	8.50 [3.50–11.30]	8.93 [5.98–11.59]	2.47 [1.17–4.33]
Pyrazinamide	109 (95.6)	88 (95.7)	21 (95.5)
Duration, months	1.70 [0.87–6.70]	2.07 [0.93–7.88]	1.00 [0.33–1.47]
Rifampicin	113 (99.1)	92 (100.0)	21 (95.5)
Duration, months	1.17 [0.63–2.13]	1.22 [0.63–2.23]	1.00 [0.67–1.53]
Aminoglycoside	22 (19.3)	17 (18.5)	5 (22.7)
Duration, months	0.88 [0.40–2.48]	1.03 [0.53–2.57]	0.37 [0.27–1.07]
Cycloserine	50 (43.9)	41 (44.6)	9 (40.9)
Duration, months	4.38 [0.95–8.85]	6.80 [0.90–10.30]	1.30 [1.23–1.70]
P-aminosalicylic acid	16 (14.0)	12 (13.0)	4 (18.2)
Duration, months	1.68 [0.54–11.32]	7.32 [0.82–13.40]	0.83 [0.38–1.27]
Prothionamide	19 (16.7)	16 (17.4)	3 (13.6)
Duration, months	1.70 [0.49–9.38]	4.60 [0.43–9.93]	1.47 [0.97–1.52]

Numbers are presented as count (percentage) or median [interquartile range].

### Follow-up after favorable response

**Figure 3:**
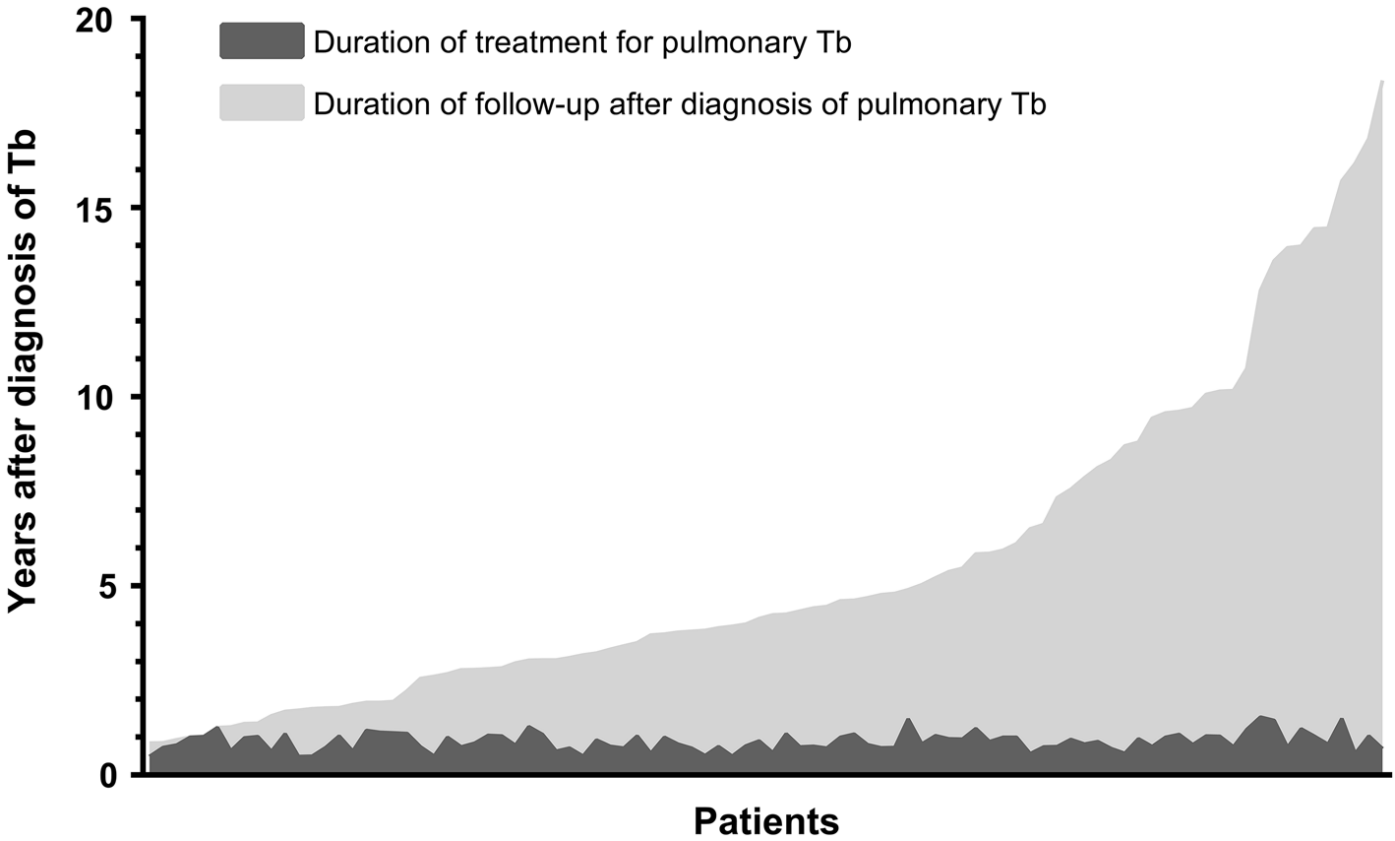
Duration of treatment and follow-ups after diagnosis of pulmonary tuberculosis for the 92 patients with favorable outcomes. The dark gray shade refers to the duration of treatment for pulmonary tuberculosis, whereas the light gray shade refers to the duration of the follow-up. The median duration of follow-up after treatment completion is 3.4 (interquartile range 1.8–6.8) years. Abbreviations: Tb, tuberculosis.

For the 92 patients with an initially favorable response, the recent health status (within 6 months) of 47 (51.1%) patients was obtained from their electronic medical records. The remaining 45 (48.9%) patients were contacted by telephone, and 22 (23.9%) responded. Three patients (3.3%) had no follow-up visits or telephone responses. The median duration of follow-up, including the telephone survey, was 3.4 (IQR 1.8–6.8) years after treatment completion (Fig. 3). Two patients (2.2%) experienced recurrence 8.5 and 65.5 months after treatment completion, respectively. One patient experienced recurrence of drug-susceptible tuberculosis, while another one experienced tuberculous empyema without known resistance patterns. The details of the two patients are presented in Supplementary Table 5.

## Discussion

In this real-world report, we evaluated the treatment regimen, initial treatment response, and follow-up outcomes in patients with drug-susceptible pulmonary tuberculosis who had to omit rifampicin because of adverse reactions. The proportion of patients with a favorable response was 80.7%, most of whom were younger with fewer comorbidities and had mild extent of radiographic involvement compared with those with an unfavorable response. In patients with a favorable response, the median duration of treatment was 10.2 months, with a four-drug or three-drug regimen consisting of first-line drugs with or without fluoroquinolones. The recurrence rate was 2.2% with a median follow-up of 3.4 years. To the best of our knowledge, this is the largest study to report the real-world experience of rifampicin-sparing treatment for drug-susceptible tuberculosis with a long-term follow-up assessment.

The high rate of the favorable response (80.7%) in our study is comparable to that achieved with regimens evaluated in recent studies for rifampicin-resistant tuberculosis, such as STREAM stage 2 (83%),^10^ MDR-END (75.0%),^8^ and the NExT study (51.0–75.0%).^11^ Although data are scarce, a few studies have reported the consequences of rifampicin-sparing treatment owing to its adverse reactions. A previous study by Park et al. analyzed patients who could not tolerate rifampicin because of adverse reactions;^25^ in their study, 20 of 25 patients (80.0%) achieved treatment success after a median of 371 days of treatment. In another study, Gupta and colleagues studied renal transplant patients who received rifampicin-sparing treatment.^26^ The study showed favorable responses in 60 of 67 (93.7%) patients after a median treatment time of 12 months, and one patient experienced recurrence.

Patients included in our study had a relatively shorter duration of treatment (median 10.2 months) compared to that in previous studies, but with a similar favorable response rate. These findings align with recent efforts to reduce the duration of treatment for drug-susceptible pulmonary tuberculosis^27,28^. Although examining the reason for this encouraging result is beyond the scope of this study, we suggest some explanations with caution. First, rifampicin was used at least once in all 92 patients with a favorable response, for a median duration of 1.22 (IQR 0.63–2.23) months. Rifampicin is highly bactericidal and effective against *M. tuberculosis* populations, not only in the metabolic state but also in the dormant state^29^. With adequate companion drugs, rifampicin is known to exert early bactericidal activity, which results in greater decrease in tuberculosis burden in the first 2 days of treatment than in the following 12 days^30^. The action of rifampicin against *M. tuberculosis* may have affected the treatment response, although it was only partly used during the early treatment course.

Second, other fundamental anti-tuberculosis medications have been actively utilized as substitutes for rifampicin. In patients with a favorable response, isoniazid and ethambutol were generally maintained during treatment (median duration of 8.87 and 8.93 months, respectively), and pyrazinamide was used for a median of 2.07 months, which is the recommended duration of use in the first-line treatment^22^. Other than the first-line drugs, fluoroquinolone was most actively used (91.3% of patients, median duration of 8.35 months), which was found to be comparable with the rifampicin-based regimen in a small group of renal transplant recipients^31^. The Curry International Tuberculosis Center and the California Department of Health also recommended a treatment regimen comprising isoniazid, ethambutol, and fluoroquinolone for 12–18 months and pyrazinamide for 2 months in cases of rifampicin-resistant tuberculosis^32^. In our study, cycloserine was also commonly used in patients with a favorable response (44.6%, median duration of 6.8 months). Cycloserine is a bacteriostatic antibiotic that inhibits cell-wall biosynthesis^33^. It is known to be efficacious against MDR tuberculosis and is currently included in category B for the treatment of MDR tuberculosis^7^.

Third, regimens consisting of at least three-drug combinations were favored. For those with a favorable response, 88 of 92 (95.7%) patients used ≥ 3 drug combinations during the intensive phase, while 81 of 92 (88.0%) patients used ≥ 3 drug combinations during the consolidation phase. This is in line with the principle of tuberculosis treatment, which requires preferentially three efficient drugs for a prolonged duration^34^.

Regarding the risk of recurrence, the results of our study should be interpreted with caution. There were no predefined follow-up protocols in our study. Although the median follow-up was 3.4 (IQR 1.8–6.8) years for 92 patients with a favorable response, 15 (16.3%) of them had < 1 year of follow-up after treatment completion. This can lead to some concern of possible recurrence, which usually occurs within a year of treatment completion. ^35^ Previous well-designed clinical trials (REMOX, OFLOTUB, and RIFAQUIN) have revealed higher rates of recurrence with shorter regiments compared to the standard regimen^36–38^. However, the findings of our study reflect our real-world practice, and every effort was made to assess recent health status for each patient.

This study has some limitations. First, the recurrence of tuberculosis was screened via electronic medical records or telephonic contact. This could have omitted patients with subclinical tuberculosis. Attention should be paid to the possible acquisition of rifampicin resistance, considering the short-term use of rifampicin^39,40^. Second, anti-tuberculosis regimens were frequently altered by the attending physician during the treatment course. Nonetheless, we have undertaken a thorough medical records review and provided detailed information regarding each drug used. Third, as a retrospective study, there were no pre-established protocols for defining adverse reactions, re-challenging drugs, or follow-up intervals. Nevertheless, we have incorporated long-term follow-up data on numerous patients to address some of these limitations.

In conclusion, we report a real-world experience of adverse reactions-necessitated rifampicin-sparing treatment for drug-susceptible pulmonary tuberculosis. Over 80% of the patients achieved an initial favorable response. They were treated with a four- or three-drug regimen for a median of 10.2 months, which included rifampicin for a median of 1.2 months. Considering the recurrence rate observed in this study (2.2% during a median follow-up of 3.4 years), attention should be paid to the patient health condition after treatment completion. We cannot make a specific recommendation based on our observations, but patients who cannot tolerate rifampicin due to adverse reactions may benefit from a shorter regimen, rather than a long-term treatment targeted for MDR tuberculosis. Future investigations involving immunomodulatory treatments for tuberculosis could potentially benefit patients experiencing adverse reactions to anti-tuberculosis medications^41^.

## Supplementary Information

Supplementary Information.

## Data Availability

The datasets used and/or analyzed during the current study are available from the corresponding author on reasonable request.
