# Supplementary material for: Real-world experience of adverse reactions-necessitated rifampicin-sparing treatment for drug-susceptible pulmonary tuberculosis
Source: Sci Rep. 2023 Jul 12;13:11275. doi: 10.1038/s41598-023-38394-1 (PMC10338469; doi:10.1038/s41598-023-38394-1)
Supplement: Supplementary file 1 — Supplementary Information. [file 41598_2023_38394_MOESM1_ESM.pdf]

Supplemental Materials for:

Real-world experience of adverse reactions-necessitated rifampicin-sparing treatment for drug-susceptible pulmonary tuberculosis

Hyung-Jun Kim, Ye-Jin Lee, Myung Jin Song, Byoung Soo Kwon, Yeon Wook Kim, Sung Yoon Lim, Yeon-Joo Lee, Jong Sun Park, Young-Jae Cho, Choon-Taek Lee, and Jae Ho Lee

Supplementary Appendix 1. Anti-tuberculosis drugs checked for susceptibility and their reference concentration

Isoniazid (0.2μg/ml)

Rifampicin (40μg/ml)

Streptomycin (10μg/ml)

Ethambutol (2.0μg/ml)

Kanamycin (30μg/ml)

Capreomycin (40μg/ml)

Prothionamide (40μg/ml)

Cycloserine (30μg/ml)

P-Aminosalicylic Acid (1.0μg/ml)

Ofloxacin (2.0–4.0μg/ml)*

Moxifloxacin (1.0μg/ml) †

Amikacin (30μg/ml)†

Levofloxacin (2.0μg/ml) †

Rifabutin (20μg/ml) †

Linezolid (2.0μg/ml) ‡

*The threshold for ofloxacin resistance changed from 2.0 to 4.0μg/ml in January 2016.

† DST testings of Moxifloxacin, Amikacin, Levofloxacin, and Rifabutin were performed after March 2010.

‡Linezolid DST testing was performed after January 2017.

Supplementary Appendix 2. STROBE Statement—Checklist of items that should be included in reports of *cohort studies*

	Item No	Recommendation	Page No
Title and abstract	1	(*a*) Indicate the study's design with a commonly used term in the title or the abstract	1
		(*b*) Provide in the abstract an informative and balanced summary of what was done and what was found	2
Introduction			
Background/rationale	2	Explain the scientific background and rationale for the investigation being reported	2–3
Objectives	3	State specific objectives, including any prespecified hypotheses	4
Methods			
Study design	4	Present key elements of study design early in the paper	4
Setting	5	Describe the setting, locations, and relevant dates, including periods of recruitment, exposure, follow-up, and data collection	4–5
Participants	6	(*a*) Give the eligibility criteria, and the sources and methods of selection of participants. Describe methods of follow-up	4–5
		(*b*) For matched studies, give matching criteria and number of exposed and unexposed	NA
Variables	7	Clearly define all outcomes, exposures, predictors, potential confounders, and effect modifiers. Give diagnostic criteria, if applicable	5–6
Data sources/ measurement	8*	For each variable of interest, give sources of data and details of methods of assessment (measurement). Describe comparability of assessment methods if there is more than one group	5–6
Bias	9	Describe any efforts to address potential sources of bias	NA
Study size	10	Explain how the study size was arrived at	4
Quantitative variables	11	Explain how quantitative variables were handled in the analyses. If applicable, describe which groupings were chosen and why	7
Statistical methods	12	(*a*) Describe all statistical methods, including those used to control for confounding	7
		(*b*) Describe any methods used to examine subgroups and interactions	7
		(*c*) Explain how missing data were addressed	NA
		(*d*) If applicable, explain how loss to follow-up was addressed	7

		(*e*) Describe any sensitivity analyses	NA
Results			
Participants	13*	(a) Report numbers of individuals at each stage of study—eg numbers potentially eligible, examined for eligibility, confirmed eligible, included in the study, completing follow-up, and analysed	7
		(b) Give reasons for non-participation at each stage	7
		(c) Consider use of a flow diagram	Fig1
Descriptive data	14*	(a) Give characteristics of study participants (eg demographic, clinical, social) and information on exposures and potential confounders	7–8
		(b) Indicate number of participants with missing data for each variable of interest	NA
		(c) Summarise follow-up time (eg, average and total amount)	9–10
Outcome data	15*	Report numbers of outcome events or summary measures over time	7–10
Main results	16	(*a*) Give unadjusted estimates and, if applicable, confounder-adjusted estimates and their precision (eg, 95% confidence interval). Make clear which confounders were adjusted for and why they were included	NA
		(*b*) Report category boundaries when continuous variables were categorized	NA
		(*c*) If relevant, consider translating estimates of relative risk into absolute risk for a meaningful time period	NA
Other analyses	17	Report other analyses done—eg analyses of subgroups and interactions, and sensitivity analyses	NA
Discussion			
Key results	18	Summarise key results with reference to study objectives	10
Limitations	19	Discuss limitations of the study, taking into account sources of potential bias or imprecision. Discuss both direction and magnitude of any potential bias	13
Interpretation	20	Give a cautious overall interpretation of results considering objectives, limitations, multiplicity of analyses, results from similar studies, and other relevant evidence	11–13
Generalisability	21	Discuss the generalisability (external validity) of the study results	13
Other information			

Funding	22	Give the source of funding and the role of the funders for the present study and, if applicable, for the original study on which the present article is based	20

*Give information separately for exposed and unexposed groups.

Note: An Explanation and Elaboration article discusses each checklist item and gives methodological background and published examples of transparent reporting. The STROBE checklist is best used in conjunction with this article (freely available on the Web sites of PLoS Medicine at http://www.plosmedicine.org/, Annals of Internal Medicine at http://www.annals.org/, and Epidemiology at http://www.epidem.com/). Information on the STROBE Initiative is available at http://www.strobe-statement.org.

Supplementary Table 1. Factors associated with a favorable response to treatment

Variables	Odds ratio	Adjusted odds ratio
Age, years	0.95 (0.91–0.98) *	0.94 (0.90–0.99) *
Female sex	1.79 (0.64–5.01)	
BMI, kg/m^2	1.05 (0.89–1.24)	
Comorbidities		
Hypertension	0.46 (0.17–1.24)	
Diabetes	0.28 (0.10–0.78) *	0.56 (0.15–2.02)
History of tuberculosis	0.88 (0.26–2.96)	
Malignancy	0.74 (0.39–1.40)	
Chronic lung disease‡	1.73 (0.20–14.83)	
Chronic liver disease	0.78 (0.62–0.99) *	0.05 (0.01–0.60) *
Chronic kidney disease	0.41 (0.19–0.89) *	0.08 (0.01–1.09)
Radiographic features		
Bilateral involvement	0.24 (0.08–0.70) *	0.46 (0.13–1.61)
Presence of cavities	0.23 (0.09–0.61) *	0.23 (0.07–0.75) *

*P-value less than 0.05. ‡Chronic lung disease refers to asthma, chronic obstructive pulmonary disease, and idiopathic pulmonary fibrosis. Adjusted odds ratio was calculated using variables of age, underlying diabetes, chronic liver disease, chronic kidney disease, bilateral involvement, and presence of cavities.

Supplementary Table 2. Detailed representative treatment regimen initially used for the treatment of pulmonary tuberculosis

Variables	Overall<br>N = 114	Favorable response<br>n = 92	Unfavorable response<br>n = 22
HREZ	106 (93.0)	84 (93.3)	22 (100.0)
HRE	3 (2.6)	3 (3.3)	0 (0.0)
E + Q CS	2 (1.8)	2 (2.2)	0 (0.0)
HRE + Q	1 (0.9)	1 (1.1)	0 (0.0)
HRZ	1 (0.9)	1 (1.1)	0 (0.0)
E + Q AG CS	1 (0.9)	1 (1.1)	0 (0.0)

Numbers indicate count (percentage).

Abbreviations: H, isoniazid; E, ethambutol; Z, pyrazinamide; Q, fluoroquinolone; CS, cycloserine; AG, aminoglycoside.

Supplementary Table 3. The detailed representative treatment regimen for the intensive phase

Variables	Overall<br>N = 114	Favorable response<br>n = 92	Unfavorable response<br>n = 22
HEZQ	25 (21.9)	23 (25.0)	2 (9.1)
HEQ	22 (19.3)	18 (19.6)	4 (18.2)
HREZ	11 (9.6)	10 (10.9)	1 (4.5)
HEQ + CS	7 (6.1)	6 (6.5)	1 (4.5)
HEZ	7 (6.1)	7 (7.6)	0 (0.0)
Q + CS PTH	4 (3.5)	4 (4.3)	0 (0.0)
EQ + AG CS	3 (2.6)	2 (2.2)	1 (4.5)
EQ + CS	3 (2.6)	1 (1.1)	2 (9.1)
HRE	3 (2.6)	2 (2.2)	1 (4.5)
HQ	2 (1.8)	2 (2.2)	0 (0.0)
HQ + AG CS	2 (1.8)	1 (1.1)	1 (4.5)
HRZ	2 (1.8)	2 (2.2)	0 (0.0)
HZQ	2 (1.8)	1 (1.1)	1 (4.5)
EQ + CS PAS	2 (1.8)	2 (2.2)	0 (0.0)
HE	2 (1.8)	2 (2.2)	0 (0.0)
HEQ + CS PAS	2 (1.8)	1 (1.1)	1 (4.5)
AG CS PTH PAS	1 (0.9)	1 (1.1)	0 (0.0)
EQ	1 (0.9)	0 (0.0)	1 (4.5)
EQ + AG	1 (0.9)	0 (0.0)	1 (4.5)
EQ + CS Lzd	1 (0.9)	0 (0.0)	1 (4.5)
EZQ + CS	1 (0.9)	1 (1.1)	0 (0.0)
EZQ + CS PTH	1 (0.9)	0 (0.0)	1 (4.5)
H + CS PTH PAS	1 (0.9)	0 (0.0)	1 (4.5)

HE + CS PTH	1 (0.9)	1 (1.1)	0 (0.0)
HEQ + AG	1 (0.9)	1 (1.1)	0 (0.0)
HEQ + AG CS PTH	1 (0.9)	1 (1.1)	0 (0.0)
HEZQ + CS	1 (0.9)	1 (1.1)	0 (0.0)
HEZQ + SXT	1 (0.9)	1 (1.1)	0 (0.0)
HQ + PAS	1 (0.9)	1 (1.1)	0 (0.0)
HREQ	1 (0.9)	0 (0.0)	1 (4.5)
HZQ + CS	1 (0.9)	0 (0.0)	1 (4.5)

Regimens persisting for ≥2 months during the early phase of treatment were defined as representative intensive phase regimens. Numbers indicate count (percentage).

Abbreviations: H, isoniazid; E, ethambutol; Z, pyrazinamide; Q, fluoroquinolone; CS, cycloserine; PTH, prothionamide; AG, aminoglycoside; Lzd, linezolid; SXT, sulfamethoxazole.

Supplementary Table 4. The detailed representative treatment regimen for the consolidation phase

Variables	Overall<br>N = 114	Favorable response<br>n = 92	Unfavorable response<br>n = 22
HEQ	26 (22.8)	21 (22.8)	5 (22.7)
HEZQ	21 (18.4)	19 (20.7)	2 (9.1)
HEQ + CS	9 (7.9)	8 (8.7)	1 (4.5)
HEZ	6 (5.3)	5 (5.4)	1 (4.5)
HQ	6 (5.3)	6 (6.5)	0 (0.0)
Q + CS PTH	5 (4.4)	5 (5.4)	0 (0.0)
EQ + CS	4 (3.5)	2 (2.2)	2 (9.1)
HZQ	4 (3.5)	3 (3.3)	1 (4.5)
HE	3 (2.6)	3 (3.3)	0 (0.0)
HZQ + CS	3 (2.6)	2 (2.2)	1 (4.5)
EQ + AG CS	2 (1.8)	1 (1.1)	1 (4.5)
H + CS PTH PAS	2 (1.8)	1 (1.1)	1 (4.5)
HE + CS	2 (1.8)	2 (2.2)	0 (0.0)
HEQ + AG	2 (1.8)	2 (2.2)	0 (0.0)
HEQ + CS PAS	2 (1.8)	1 (1.1)	1 (4.5)
EQ	1 (0.9)	0 (0.0)	1 (4.5)
EQ + AG	1 (0.9)	0 (0.0)	1 (4.5)
EQ + CS Lzd	1 (0.9)	0 (0.0)	1 (4.5)
EQ + CS PAS	1 (0.9)	1 (1.1)	0 (0.0)
EQ + PTH PAS	1 (0.9)	1 (1.1)	0 (0.0)
EZQ + CS	1 (0.9)	1 (1.1)	0 (0.0)
EZQ + CS PTH	1 (0.9)	0 (0.0)	1 (4.5)
HEQ + CS → HEQ*	1 (0.9)	1 (1.1)	0 (0.0)

HEQ + PTH	1 (0.9)	1 (1.1)	0 (0.0)
HEZ + SXT → HEZ*	1 (0.9)	1 (1.1)	0 (0.0)
HQ + AG CS	1 (0.9)	0 (0.0)	1 (4.5)
HQ + CS PAS	1 (0.9)	1 (1.1)	0 (0.0)
HQ + PAS	1 (0.9)	1 (1.1)	0 (0.0)
HRE → HEQ*	1 (0.9)	1 (1.1)	0 (0.0)
HREQ	1 (0.9)	0 (0.0)	1 (4.5)
HZ	1 (0.9)	1 (1.1)	0 (0.0)
Q + CS PAS	1 (0.9)	1 (1.1)	0 (0.0)

Regimens persisting ≥2 months after the intensive phase of treatment were defined as representative consolidation phase regimens. Numbers indicate count (percentage). *Indicates patients who changed their consolidation regimen during treatment; both regimens were used for ≥2 months.

Abbreviations: H, isoniazid; E, ethambutol; Z, pyrazinamide; Q, fluoroquinolone; CS, cycloserine; PTH, prothionamide; AG, aminoglycoside; Lzd, linezolid; SXT, sulfamethoxazole.

Supplementary Table 5. Details of the two patients who experienced recurrence of pulmonary tuberculosis after treatment completion

Variables	Patient 1	Patient 2
Age, years	77	55
Sex	Female	Male
Comorbidity	Asthma	History of tuberculosis
Type of adverse reaction to rifampicin	Fever	Skin rash / Pruritus, Hepatotoxicity
Initial radiographic findings		
Cavity	No	Yes
Bilateral involvement	Yes	Yes
Treatment regimen		
Total duration of treatment[a]	7.7 months	18.2 months
Initial regimen	HREZ	HREZ
Intensive phase regimen	HEZQ	EQ + CS PAS
Consolidative phase regimen	HEZQ	Q + CS PAS
Duration of R[b]	0.1 months	3.9 months
Duration of H[b]	5.8 months	4.7 months
Duration of Z[b]	4.4 months	0.5 months
Duration of E[b]	5.7 months	14.4 months
Duration of Q[b]	5.5 months	17.7 months
Drug resistance after recurrence	Susceptible to all drugs	Unknown (tuberculous empyema)
Clinical outcome		
Time to recurrence	8.5 months after treatment completion	65.6 months after treatment completion
Death	Yes	No
Time to death	20.5 months after treatment completion	-
Cause of death	Myelodysplastic syndrome	-

[a]The overall treatment duration was calculated by counting the calendar days from the first day of treatment to the last. [b]Duration of each drug were calculated as actual dosage days prescribed.

Abbreviations: H, isoniazid; R, rifampicin; E, ethambutol; Z, pyrazinamide; Q, fluoroquinolone; CS, cycloserine; PAS, p-aminosalicylic acid.
